# Spheres, tears, and spears: Regulating the perimeter and circularity of millimeter-sized alginate hydrogel beads

**DOI:** 10.1002/aic.70279

**Published:** 2026-02-10

**Authors:** Conor G. Harris, Katherine D. Bandettini, Hannah K. Gedde, Lewis Semprini, Kaitlin C. Fogg, Willie E. Rochefort

**Affiliations:** School of Chemical, Biological, and Environmental Engineering, Oregon State University, Corvallis, Oregon, USA

**Keywords:** dispensing, fluid mechanics, interfacial dynamics, multiphase flow, rheology

## Abstract

Generating hydrogel beads pertains to many engineering applications. We examined two alginate-based fluids at three concentrations of alginate, $c_{\text{AG}}$. We used the “Map of Misery” to determine which material property (viscosity, elasticity, and inertia) drives droplet formation. Next, we measured the perimeter and circularity of hydrogel beads as a function of nozzle radius and velocity to generate an operating space that identifies the parameters necessary to form spherical, monodisperse millimeter-sized hydrogel beads (*spheres*) for all $c_{\text{AG}}$. Further, we observe interactions between $c_{\text{AG}}$ and Ca that resulted in an increase of the slope between perimeter and Capillary number (Ca), and *tear* and *spear* shapes of hydrogel beads. Lastly, we corroborated the value of the jet velocity used for the dimensionless groups with high-speed imaging and computational data of the interfacial flow maintained during drop formation. Computations were performed using the open-source computational fluid dynamics software, OpenFOAM.

## 1 | INTRODUCTION

Controlled dispensing of complex fluids containing polysaccharide or carbohydrate polymers pertains to many applications.^1,2^ This includes the generation of millimeter-sized hydrogel beads used for applications such as: bioprinting,^3^ drug delivery,^4,5^ food gels,^6^ cell immobilization,^7,8^ and bioremediation.^9–13^ Dispensing polysaccharide pre-gel solutions allows users to tune the size and shape of hydrogel beads, which controls the rate of diffusion and mechanical properties (e.g., the compressive and tensile strength).^14–16^ However, these precursor gel solutions exhibit non-Newtonian or viscoelastic behavior, and there is limited mechanistic research on their dispensing processes.^17^ Thus, instruments are calibrated for individual solutions to generate hydrogel beads, which can be time intensive and lead to errors when operating conditions change.

**FIGURE 1:**
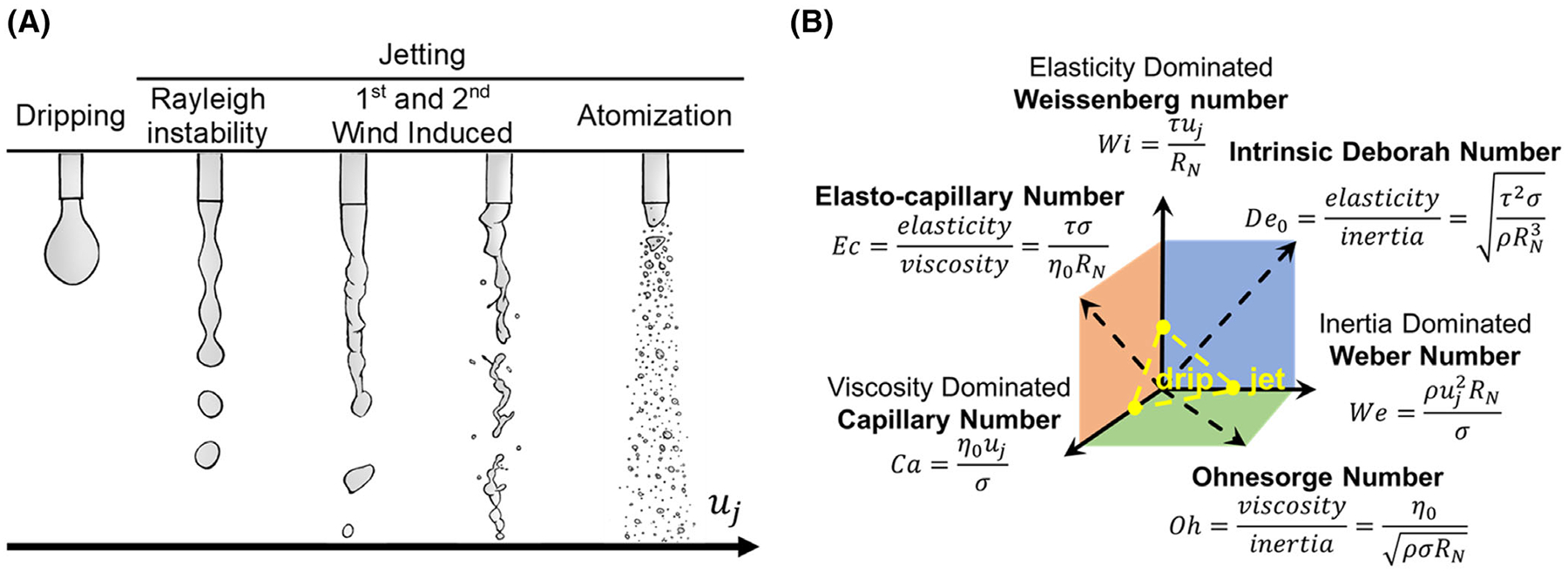
(A) Flow regimes of fluid dispensing as a function of jet velocity, $u_{j}$. Dispensing is led by dripping and eventually moves into four distinct jetting regimes: Plateau-Rayleigh instability, first and second wind induced, and atomization. (B) Diagram of the Map of Misery.^17^

The dispensing of solutions can be classified as either a *dripping* or a *jetting* regime based on the nozzle exit velocity, or jet velocity, $u_{j}$ (Figure 1A).^18,19^ Under the dripping regime, single droplets form at the nozzle tip and detach due to gravity once a critical volume is reached. As $u_{j}$ increases, the liquid enters a jetting regime where a continuous jet or filament is maintained after ejecting from the nozzle. Four distinct regimes occur in the jetting regime as $u_{j}$ increases: *Plateau-Rayleigh instability, first wind-induced, second wind-induced*, and *atomization*. The Plateau-Rayleigh instability regime generates spherical droplets that are smaller in diameter than droplets formed in the dripping regime. The droplets occur from a sinusoidal perturbation in the filament generated by competing surface tension forces and material properties that resist thinning (i.e., inertia, viscosity, and/or elasticity). Both wind-induced regimes are characterized by a chaotic filament with non-spherical droplets of the same size as Plateau-Rayleigh. Lastly, the atomization regime is a spray of fluid droplets that range between 50 and 100μm in diameter. Due to the irregular droplets formed in the wind-induced regimes, and the range of size obtained from atomization, only the dripping and Plateau-Rayleigh regimes were deemed suitable for the generation of millimeter-sized hydrogel beads in this work.

Technology, such as coaxial air devices and piezoelectric oscillators used to vibrate the dispensing nozzle, has been shown to modify the size and shape of micrometer-sized droplets.^20–22^ However, in a preliminary study using the statistical optimization tool, Design of Experiments, we discovered that both coaxial air flow and oscillator frequency had little effect on the size and shape of millimeter-sized droplets formed when compared to the fluid flow (Figure S1). Therefore, we focused solely on fluid flow parameters throughout this study.

Material properties and operating conditions control fluid filament thinning. The extension of a fluid filament requires the filament to thin, which eventually leads to a breakup and generates fluid droplets. In addition to the surface tension of the fluid, the material properties, inertia (density), viscosity, elasticity (relaxation time), or a combination of these properties resist the thinning of a filament in extension. Thus, a guide to dispensing fluids, named Map of Misery,^17^ characterizes fluids with nondimensional numbers consisting of the density, viscosity, relaxation time, and surface tension of the fluid (Figure 1B). Comparisons between the nondimensional numbers of the fluids to critical transition criteria identify which material property dominates the thinning of the filament of a dispensing fluid. With knowledge of the appropriate material property that dominates the thinning, the radius of the filament as a function of time can be modeled accurately.

As far as we know, the use of this map to characterize hydrogel precursor solutions and evaluate the size and shape of hydrogel beads with those results has not been conducted. For instance, many papers suggest that the Weber number, which compares the inertial and surface tension forces, can be used to characterize flow parameters based on ink-jet printing applications.^23^ However, the Weber number may prove inadequate for entangled polymer solutions with higher viscosities. Further, the literature on dispensing hydrogel precusor solutions has suggested using rainfall models or high-speed imaging to calculate $u_{j}$, which requires users to calibrate their instruments with high-speed imagery. Therefore a consistent and simple method to determine or set $u_{j}$ would reduce the need to calibrate instruments. In this work, we explore the effects of a wide range of experimental parameters, nozzle sizes, flow conditions, and pre-gel solution concentrations that generate *sphere, tear, and spear* shaped hydrogel beads. Lastly, we compare droplet formation captured using high-speed imaging with a numerical simulation performed in OpenFOAM to corroborate the values of $u_{j}$ used in this study.

## 2 | MATERIALS AND METHODS

### 2.1 | Model fluid constituents and preparation

Two model fluids were used throughout this study to compare the effects of additives to hydrogel precursor solutions (Table 1). The primary constituent used to generate hydrogel beads was the polysaccharide, sodium alginate (Cape Crystal Brands, Summit, New Jersey, USA), well known for its use in generating hydrogels for a range of different applications. For all solutions, the concentrations of sodium alginate were $c_{\text{AG}}\in \left\{1,1.5,2\right\}\%\;\text{w}/\text{v}$. The solutions constituents and details are provided below:

1. An emulsion used in the generation of hydrogel beads with applications in bioremediation. Tetrabutyl orthosilicate (TBOS) (United Chemical Technologies, Levittown, Pennsylvania, USA), a slow-release compound used to maintain microbial activity for select microorganisms, such as *Rhodococcus rhodochrous* American Type Culture Collection (ATCC) 21198,^10–12^ was added at 4.5% w/v. To properly emulsify the solutions, Span80 (Ohio Valley Specialty Company, Marietta, Ohio, USA), a non-ionic surfactant, was added at 0.1% w/v. The remainder of the solution was comprised of de-ionized water, with concentrations dependent on $c_{\text{AG}}$.
2. A suspension of titanium dioxide (Sigma-Aldrich, St. Louis, Missouri, USA), at a concentration of 0.1% w/v, in sodium alginate hydrogel precursor solutions. This solution represented standard sodium alginate hydrogels without the presence of a surfactant. Suspended titanium dioxide aided in the imaging of hydrogels, extensional rheology, and filament breakup measured with high-speed imaging. The remainder of the solution was comprised of de-ionized water, with concentrations dependent on $c_{\text{AG}}$.

### 2.2 | Fluid characterization

#### 2.2.1 | Steady-shear experiments to measure viscosity of solutions

Steady-shear rheological measurements were performed on an ARG2 rheometer (TA instruments, New Castle, Delaware, USA) with a 60 mm 1° cone top geometry and a Peltier plate bottom geometry. Steady shear experiments were performed on model fluid solutions to determine the viscosity of solutions with respect to shear rate. Samples were loaded and subjected to a logarithmic shear rate ramp, $\dot{\gamma }=0.1-500{\;\text{s}}^{-1}$. The resultant stress was measured as a function of shear rate, $\dot{\gamma }$, and viscosity, η, was determined by the ratio of stress to shear rate.

#### 2.2.2 | Extensional rheology from a dripping onto substrate method to measure extensional relaxation times

Extensional rheology was performed using a dripping-onto-substrate (DOS) method.^24^ In this method, a stretched liquid bridge is formed between a sessile drop and nozzle and the liquid bridge, or filament, is tracked via high-speed imaging. Solutions were dispensed through a nozzle (outer diameter, $2R_{O}=1.27\;\text{mm}$) onto a glass slide at a height $\left(H/2R_{O}\right)$ approximately equal to 3. The volumetric flow rate was maintained at $\dot{V}=0.02\;\text{mL}{\;\text{min}}^{-1}$ with an IPS-14S syringe pump (Inovenso Technology Inc., Cambridge, Massachusetts, USA). High-speed footage was captured using a Chronos 1.4 high-speed camera (Kron Technologies, Burnaby, British Colombia, Canada) with a frame rate of 8800 frames per second (FPS).

### 2.3 | Generating hydrogel beads with the bead generator

**FIGURE 2:**
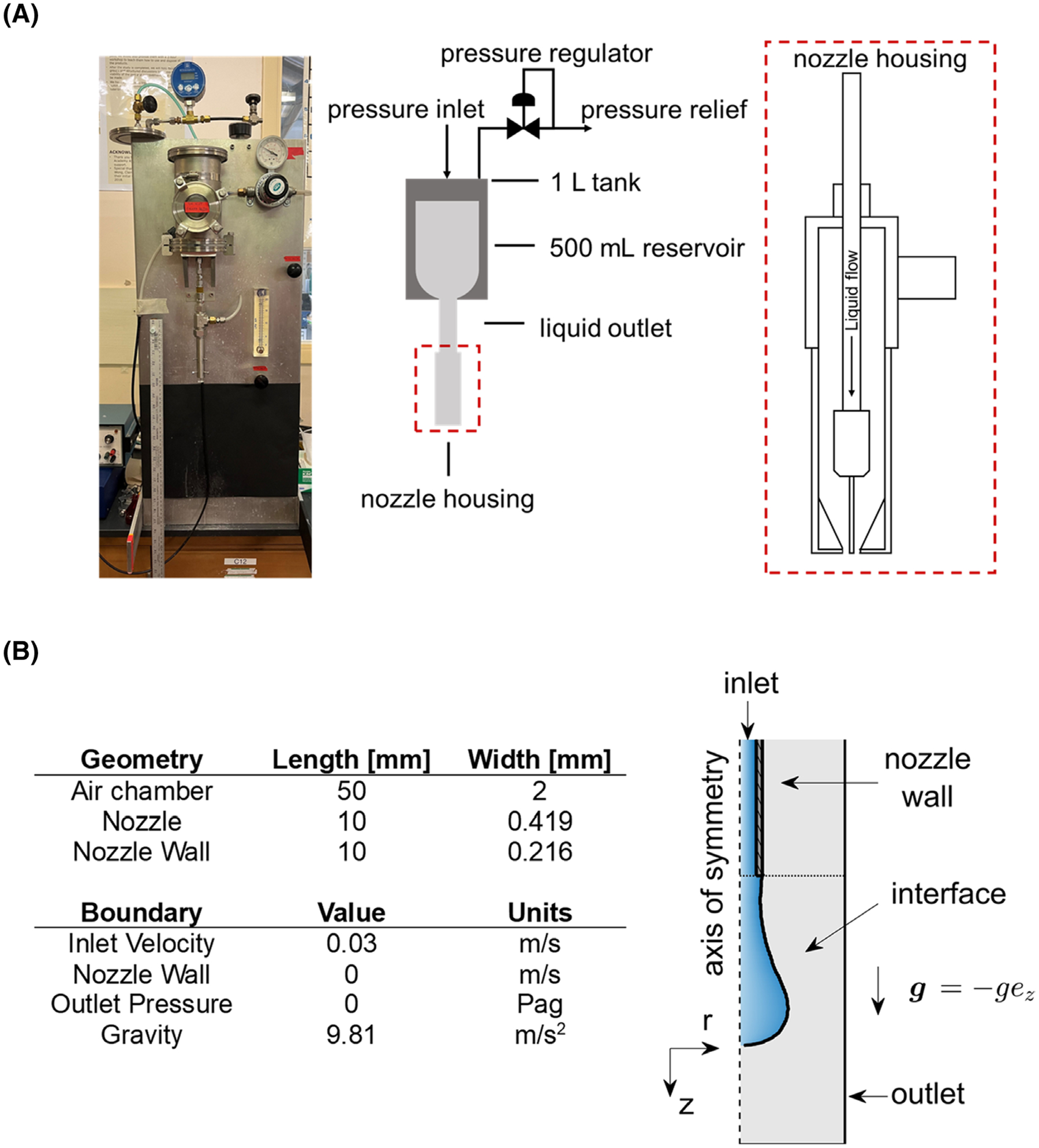
(A) Schematic and photo of the bead generator. (B) Flow domain, geometry, and boundary conditions used in the bead generator and high-speed experiments, as well as numerical calculations used to simulate high-speed experiments.

A pressured tank bead generator was used to generate hydrogel beads throughout this study (Figure 2A). The bead generator consisted of a 1 L stainless steel tank with a 500 mL reservoir. The tank was equipped with an air inlet used to pressurize the tank along with a pressure relief line to control and maintain a constant pressure head. A liquid outlet connected to the reservoir was fastened below the tank for the liquid to flow. Different size capillaries were used with nozzle radii $R_{\text{N}}=0.3$, 0.4, and 0.6 mm.

To generate hydrogel beads, the bead generator tank was filled with model fluid solution, sealed, and pressurized to initiate and maintain flow. The pressure ranged from 4.1 to 192 kPa (0.6–28 pounds per square inch (PSI)) for all experiments. The operating conditions were based on the capability for solutions to flow and generate millimeter-sized droplets. The volumetric flow rate $\dot{V}$ was determined by measuring the volume of solution dispensed from the nozzle over time. The jet velocity $u_{j}$ was taken as the ratio between the volumetric flow rate and the cross-sectional area of the nozzle $A_{\text{N}}$, that is, $u_{j}=\dot{V}/A_{N}$. The model fluids were dropped into a 1.5% w/v calcium chloride solution, which instantaneously formed hydrogel beads and preserved the geometry of the falling droplet. These hydrogel beads were used for image analysis (Section 2.5).

### 2.4 | Dimensional analysis

Droplet formation occurs from the thinning of the liquid filament that is dispensed from the nozzle and the surface tension of the fluid, σ. Further, thinning is resisted by fluid properties: viscosity η, density ρ, and relaxation time τ. For all dimensionless groups with η, we use a zero-shear viscosity $\eta_{0}=\eta (\dot{\gamma }\to 0)$. In addition, we analyzed all dimensionless groups as a “global” dimensionless group, such that the filament radius was taken as the initial radius, or the radius of the nozzle, $R_{\text{N}}$. This was selected due to the difficulty of measuring the filament radius for every droplet forming measurement. One of the stated goals of this work was to simplify the experimental requirements to determine bead size, and this eliminates the need to measure the filament at any point in the process.

#### 2.4.1 | Material property dimensionless groups

The material property dimensionless groups are used to characterize the material property that dominates the resistance to thin at a given filament diameter (Figure 1B). The specific values that designate which material property dominates are derived in Clasen et al. by comparing the numerical solutions of capillary velocities $\left(U=-dR/\text{dt}\right)$ controlled by viscosity, inertia, or elasticity.^17^

The Ohnesorge number Oh compares the viscous and inertial effects for free surface flows:

(1) $$\text{Oh}=\frac{\eta_{0}}{\sqrt{\rho \sigma R_{\text{N}}}}$$

For Oh > 0.2, the viscosity controls thinning, whereas Oh < 0.2, the inertia will control the thinning.

The elasto-capillary (EC) number Ec compares the elastic and viscous effects:

(2) $$\text{Ec}=\frac{\tau \sigma }{\eta_{0}R_{\text{N}}}$$

When elasticity controls thinning, Ec > 4.7, but viscosity controls thinning when Ec < 4.7.

The intrinsic Deborah number ${\text{De}}_{0}$ is used to relate the elastic and inertial forces:

(3) $${\text{De}}_{0}=\sqrt{\frac{\tau^{2}\sigma }{\rho {R_{\text{N}}}^{3}}}$$

With $\text{D}{\text{e}}_{0}>1$, elastic effects outweigh inertia effects, and when $\text{D}{\text{e}}_{0}<1$, inertial forces dominate the thinning in comparison to elasticity.

Two of the three material property dimensionless groups are independent, with ${\text{De}}_{0}=\text{OhEc}$.

#### 2.4.2 | Dynamic dimensionless groups

The dynamic conditions represented in the Map of Misery^17^ consist of the following dimensionless groups (Figure 1B):

The capillary number Ca compares the viscous and surface tension effects:

(4) $$\text{Ca}=\frac{\eta_{0}u_{j}}{\sigma }$$

The Weber number We compares the inertial and surface tension forces:

(5) $$\text{We}=\frac{\rho u_{j}^{2}R_{\text{N}}}{\sigma }$$

Finally, the Weissenberg number Wi describes flow for elastic dominated thinning:where

(6) $$\text{Wi}=\frac{\tau u_{j}}{R_{\text{N}}}$$

These three groups were selected as they each compare the capillary velocity $\left(U=-dR/\text{dt}\right)$ to the jet velocity $u_{j}$ used to determine a transition between dripping and jetting, and are commonly used to describe the operating conditions for free surface flow.^25^ It should be noted that Wi = CaEc, and We = Ca^2^Oh^−2^. Therefore, only three out of the six dimensionless groups are independent, and fluids can be fully described with three dimensionless groups: two material property-based and one operating dimensionless group.

### 2.5 | Perimeter and circularity of beads

Hydrogel beads formed with the bead generator were imaged to characterize the size and shape of the droplets produced from the generator (Section 2.3). Beads were taken from the crosslinking bath and placed on a custom microscope stage for imaging. Images were captured on a Firefly GT305 digital microscope (Firefly Global, Belmont, MA, USA). Images were processed using MATLAB (MATLAB, Natick, MA, USA) using the Image Processing Toolbox. Beads were separated from the background image through thresholding, and area and perimeter data were calculated for nine beads $\left(n=9\right)$ (Figure S2).

### 2.6 | Filament breakup and droplet formation studied with high-speed imaging

Dripping and jet breakup effects were captured using a Cronos 1.4 High Speed Camera (Kron Technologies Inc., Burnaby, British Columbia, Canada). The jet was illuminated with a Godox SL150III Video Light (SZ Godox Technology Co., Ltd., Shenzhen, China). In order to corroborate computational modeling results (Section 2.7, below), a single model fluid $\left(c_{\text{AG},\text{TBOS}}=1.5\%\;\text{w}/\text{v}\right)$ with one nozzle radii $\left(R_{\text{N}}=0.4\;\text{mm}\right)$ was studied to evaluate filament breakup within the dripping regime and to compare velocity measured by hand. The jet velocity was maintained at $u_{j}=0.03\pm 0.007{\;\text{ms}}^{-1}$ (mean ± standard deviation), based on three replicate volumetric flow rate measurements collected during droplet formation.

The setup to collect the jet breakup video consisted of high-speed camera facing the video light with the jet in between. Therefore, the jet appeared as a dark silhouette and the background was bright light that allowed for image thresholding. To obtain the pixel size, the nozzle with a known outer diameter $D_{\text{o}}=1.3\;\text{mm}$ was captured in the video. MATLAB was used to threshold high-speed footage and measure the radius of the jet $R_{t}$ as a function of t using the Image Processing Toolbox. Time was computed based on the frame rate, fixed at 3030 FPS.

### 2.7 | Computational modeling using the solver interFoam from OpenFOAM

The numerical methods presented in this study were conducted using OpenFOAM version 9.^26^ Specifically, a volume-of-fluid (VOF) method was employed to model the multiphase fluid flow using solvers from the standard OpenFOAM library, *interFoam*. The VOF method has been used to simulate drop formation processes by others, with endorsements of the capability of VOF based on accuracy and reliability of the determination of pinch-off dynamics.^27–29^

#### 2.7.1 | Mathematical model

The mathematical model used in this study has been well documented and used to describe the flow of a liquid jet being ejected from a round nozzle by Torres and Fonte.^27^

A generalized Newtonian constitutive equation was used to model the alginate fluids:

(7) $$\tau_{\text{liquid}}=2\eta (\dot{\gamma })D$$

where $D=\frac{1}{2}\left[\nabla u+(\nabla u{)}^{T}\right]$ is the rate-of-deformation tensor and u is the velocity vector. The model equation used for the generalized Newtonian fluid was a Bird–Carreau or Carreau–Yasuda (CY) equation:^30,31^

(8) $$\eta (\dot{\gamma })=\eta_{\infty }+\frac{\eta_{0}-\eta_{\infty }}{{\left(1+{\left(K_{\text{CY}}\dot{\gamma }\right)}^{a_{\text{CY}}}\right)}^{n_{\text{CY}}-1/a_{\text{CY}}}}$$

where η is the solution viscosity, $\eta_{\infty }$ is the infinite shear viscosity, $\eta_{0}=\eta (\dot{\gamma }\to 0)$ is the zero-shear viscosity, $\dot{\gamma }$ is the shear rate, and $a_{\text{CY}},\;K_{\text{CY}}$, and $n_{\text{CY}}$ are model parameters. The CY model was selected due to its capability to capture the rheology of shear-thinning polymer solutions above the dilute concentration regime and its prevalence in modeling sodium alginate viscosity data.^32–34^

#### 2.7.2 | Flow domain and boundary conditions

The geometry and boundary conditions were designed to replicate the experimental setup described in Section 2.3, similar to the configuration used by others in the literature (Figure 2B).^17,27,28^ The geometry consisted of an axial slice of a blunt cylindrical nozzle suspended in an air chamber. Assuming the bead generator nozzle was perfectly aligned in the direction of gravity, the flow was modeled as two-dimensional (2D) and axisymmetric, such that no flow occurred into or out of the 2D plane. Although the nozzle was aligned prior to the experiments, small residual misalignments in the nozzle could lead to discrepancies in pinch-off time and droplet size between computational and experimental results. The air chamber was set to a length of 50 mm and a width of 2 mm. The nozzle radius was 0.419 mm and the nozzle wall thickness was 0.216 mm, based on an 18-gauge nozzle used in experiments. Additionally, the nozzle was designed with a length of 10 mm to allow flow to stabilize within the nozzle before exiting.

The initial and boundary conditions were based on the parameters high-speed imaging experiments (Section 2.6). The initial condition was set with the liquid phase entering the nozzle at the inlet with a velocity $u_{j}=0.03{\;\text{ms}}^{-1}$, and the air in the chamber was at rest. A no slip condition was maintained for the walls of the nozzle $\left(u=0\right)$. A zero-gauge pressure condition was used for the outlet of the domain (top, bottom, and sides of the air chamber), and thus, the air chamber was considered open to the atmosphere. Gravity was modeled to act in the z direction such that $g=9.81e_{z}\;{\text{ms}}^{-2}$. Because the fluid in experimental flows was found to maintain contact with nozzle wall at the tip (i.e., the nozzle wall parallel to the r-direction), a constant contact angle was applied at this boundary using the built-in function in OpenFOAM, *constantAlphaContactAngle*, with a contact angle $\theta =45^{\circ }$. This boundary condition ensures wetting of the nozzle tip and was found to improve agreement between computation and experimental results for the maximum droplet radius. At the symmetry line, the *symmetryPlane* boundary condition used in OpenFOAM was maintained.

The transport properties (i.e., viscosity, density, and surface tension) of the fluid dispensed at room temperature (20°C) were set based on the model fluid captured with high-speed imaging $c_{\text{AG,TBOS}}=1.5\%\text{w}/\text{v}$ (Table 2). The surrounding fluid was modeled as air, with viscosity $\mu_{\text{air}}=1.8\times 10^{-5}$ Pas and density $\rho_{\text{air}}=1.2\;\text{kg}{\;\text{m}}^{-3}$. Therefore, the viscosity ratio was $\lambda =\eta_{0}/\mu_{\text{air}}=3.3\times 10^{5}$.

#### 2.7.3 | Numerical simulations

The domain was discretized with approximately 94,800 quadrilateral cells, with a side length $\Delta x=0.08R_{\text{N}}$. The time step Δt was adjusted based on the Courant–Freidrich–Lewy (CFL) condition with a Courant number $C=u_{\text{max}}\Delta t/\Delta x\leq 0.1$, and the value was selected to increase accuracy and prevent numerical instability.^27,35^ The pressure–velocity coupling was solved with a *pressure implicit with splitting of operators* algorithm. Transient terms were discretized by a Crank Nicolson 1 scheme. The Laplacian and convective terms were discretized by a Gauss linear corrected and Gauss limited LinearV 1 schemes, respectively.^36^ In addition, the convection and compressive term in were discretized with a *multi-cut piecewise-linear interface calculation scheme*. Lastly, the *Multidimensional Universal Limiter with Explicit Solution* solver was implemented to refine the interface between the air and liquid.^37^

## 3 | RESULTS

### 3.1 | Material properties

#### 3.1.1 | Steady shear

**FIGURE 3:**
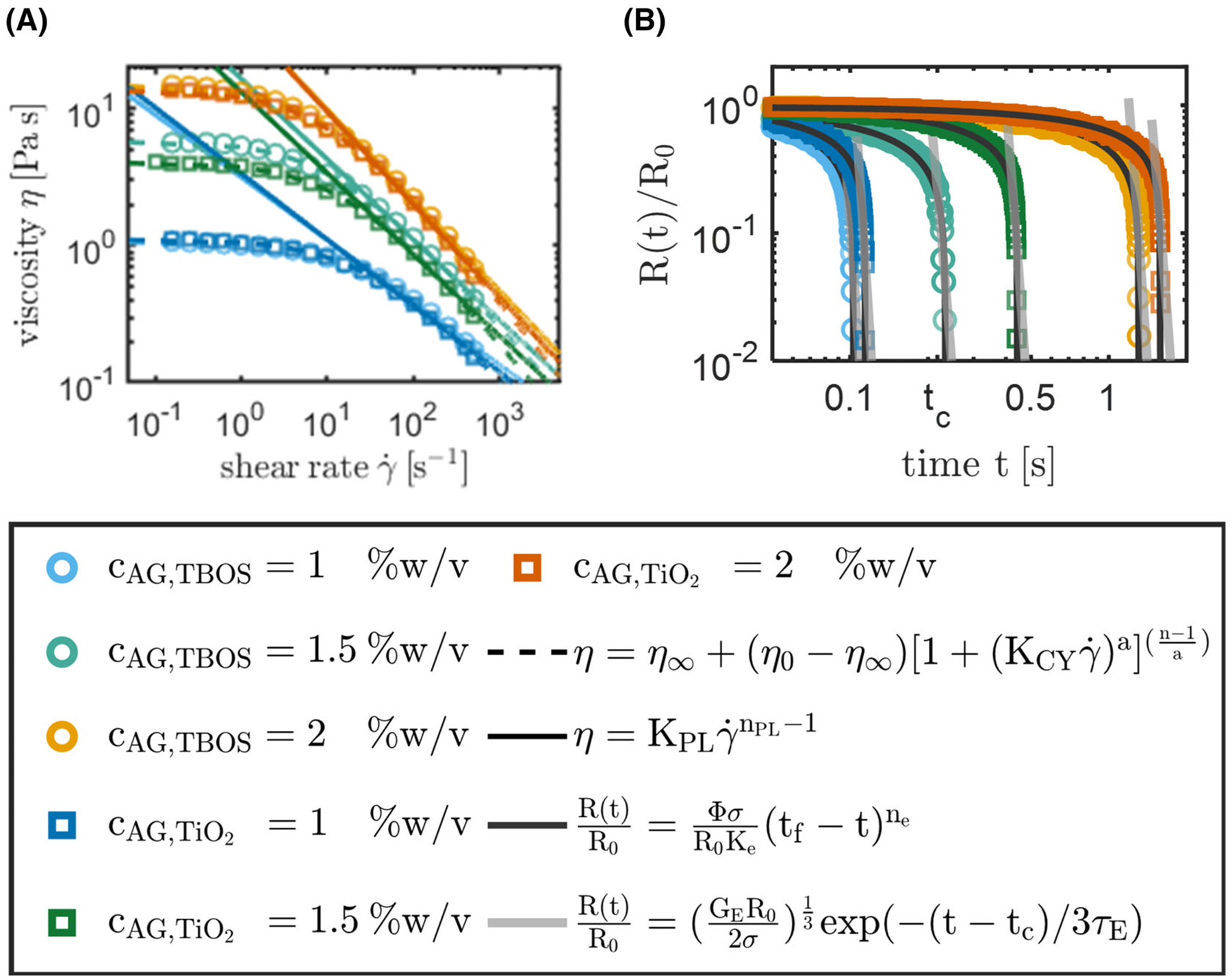
Shear and extensional rheology results used to extract material property values for alginate solutions. (A) Log–log plot of viscosity η with respect to shear rate $\dot{\gamma }$ for alginate solutions. Points consist of averaged viscosity from three replicates $\left(n=3\right)$, and error bars omitted for clarity as they lie within the points. Dotted and solid lines represent of Carreau–Yasuda and power law models, respectively. (B) Log–log plot of normalized radius $R(t)/R_{0}$ with respect to time t for alginate solutions. Points consist of single measurements $\left(n=1\right)$, representative of three replicates, as standard deviations were smaller than plotted points. Black and gray solid lines represent power law and elasto-capillary fits, respectively. AG, alginate; TBOS, tetrabutyl orthosilicate; ${\text{TiO}}_{2}$, titanium dioxide.

Steady shear flow sweeps were conducted to evaluate the viscosity dependence on shear rate for both model fluids at three different sodium alginate concentrations $c_{\text{AG}}\in \left\{1,1.5,2\right\}\%\;\text{w}/\text{v}$ (Figure 3A). The viscosity for all solutions exhibited a shear-thinning behavior with a viscosity plateau at low shear rates, or a zero-shear viscosity $\eta_{0}=\eta (\dot{\gamma }\to 0)$. Thus, viscosity data were fit using the CY model (Equation 8). For all models, $\eta_{\infty }$ was maintained as 0.001 Pas, and $a_{\text{CY}}$ was maintained as 0.77, obtained by minimizing the mean squared error between the model and experimental data. Otherwise, $\eta_{0}$ and $K_{\text{CY}}$ were fit unbounded, and $n_{\text{CY}}$ was fitbounded between 0 and 2. In addition, the value of $K_{\text{CY}\dot{\gamma }}$ can be treated as a Weissenberg number, which suggests $K_{\text{CY}}\sim \tau_{\text{s}}$, confirmed by dynamic oscillatory measurements (Figure S3).

As expected, the zero-shear viscosity $\eta_{0}=\eta (\dot{\gamma }\to 0)$ and the relaxation time $\tau_{\text{s}}$ increased with polymer concentration $c_{\text{AG}}$ (Table 2). Increases in polymer concentration increases the entanglements of polymer in solutions and increases the resistance to flow and elastic properties. Conversely, the shear-thinning index $n_{\text{CY}}$ decreased with increasing $c_{\text{AG}}$, consistent with previous studies and suggests that the entanglement density decreases more rapidly with shear, compared to the rest state, at higher concentrations.^38,39^ Rodríguez-Rivero demonstrated that alginate solutions transition from entangled polyelectrolyte behavior to entangled neutral-polymer behavior at high $c_{\text{AG}}\;\left(c_{\text{AG}}≳1\%\text{w}/\text{v}\right)$.^39^ Consequently, analogous to polymer melts, increased entanglement density promotes shear thinning. A greater fraction of polymers orient in the direction of the flow and shear thinning initiates at lower deformation rates (Wi > 1).^40^

In addition to the CY model, flow sweeps were fit with a power law (PL) model after the zero-shear plateau $\eta =\eta (\dot{\gamma }>10)$:

(9) $$\eta (\dot{\gamma })=K_{\text{PL}}{\dot{\gamma }}^{n_{\text{PL}}-1}$$

where η is the solution viscosity, $\dot{\gamma }$ is the shear rate and $K_{\text{PL}}$ and $n_{\text{PL}}$ are model parameters.

The PL model was fit to the shear-thinning region of the flow sweep to enable comparison with extensional rheology, since only PL models are currently available for describing extensional rheology for shear-thinning fluids (Section 3.1.2). In general, the model parameters followed the same trends observed with the CY model (Table 2). Note that the values for $K_{\text{PL}}$ is greater than $\eta_{0}$ as a PL model does not account for a zero-shear plateau.

#### 3.1.2 | Extensional rheology with dripping-onto-substrate

Extensional rheology from a dripping-onto-substrate (DoS) method was conducted for all model fluids. The ratio of the time-dependent radius of the filament $\left(R(t)\right)$ to the initial radius of the filament $\left(R_{0}=R(t=0)\right)$ was extracted from high-speed imagery using an in-house MATLAB script (Figure 3B). Fluids that possess rate dependent high viscosities (Oh > 1), capillary thinning undergoes PL thinning that leads to pinch-off. However, for viscoelastic fluids, an EC regime occurs after the initial thinning dynamics that delays the pinch-off time and decays exponentially. A PL model characterizes the PL regime:

(10) $$\frac{R(t)}{R_{0}}=\Phi \frac{\sigma }{K_{e}R_{0}}{\left(t_{\text{f}}-t\right)}^{n_{\text{e}}}$$

where R(t) is the radius of the filament tracked over time, $R_{0}$ is the initial radius of the filament at time $t_{0},K_{\text{e}}$ is the PL prefactor coefficient in extension that can be treated as an approximation for viscosity, σ is the surface tension determined from pendant drop measurements (Figure S4), $t_{\text{f}}$ is the pinch-off time, t is time, $n_{\text{e}}$ is the PL exponent in extension, and Φ is the prefactor constant dependent on the value of $n_{\text{e}}$.

We observed capillary thinning driven by PL dynamics, demonstrated by the excellent fit between the PL model and experimental data across all times.

We observed differences in magnitude between $K_{\text{e}}$ and $K_{\text{PL}}$, however, $K_{\text{e}}$ exhibited a similar trend to $K_{\text{PL}}$ and increased with increases in $c_{\text{AG}}$ (Table 2). $n_{\text{e}}$ decreased between $c_{\text{AG}}=1\%$ and 2% w/v similar to $n_{\text{PL}}$, but the difference between $n_{\text{e}}$ at $c_{\text{AG}}=1\%$ and 2% w/v was smaller compared to $n_{\text{PL}}$. The differences between shear and extensional rheology likely occur due to the influence of deformation history.^41^

Finally, the EC regime can be fit with the following exponential decay function:

(11) $$\frac{R(t)}{R_{0}}\sim {\left(\frac{G_{\text{E}}R_{0}}{2\sigma }\right)}^{1/3}\text{exp}\left(-\frac{t-t_{\text{c}}}{3\tau_{\text{E}}}\right)$$

where R(t) is the radius of the filament tracked over time, $R_{0}$ is the initial radius of the filament at time $t_{0},G_{\text{E}}$ is the elastic modulus, σ is the surface tension determined from pendant drop measurements (Figure S4), $t_{\text{c}}$ is the time of onset of the EC regime, and $\tau_{\text{E}}$ is the extensional relaxation time.

The EC regime was estimated at the late stages of the capillary thinning;^24^ fit similarly to previous studies which evaluated the extensional rheology of alginate solutions.^32,39^ To reduce the subjectivity of the fit, described by Rodríguez-Rivero et al.;^39^ we fit Equation (11) based on the inflection point of the derivative of the radius with respect to time, normalized by the pinch-off time and initial radius, $\left(t_{\text{f}}/R_{0}\right)(dR(t)/\text{dt})$. The results show an increase in $\tau_{\text{E}}$ with increases in $c_{\text{AG}}$, as expected. Increases in $c_{\text{AG}}$ increases the number of entanglements in these solutions, which extends the time required for polymers to relax after being perturbed.^32,42^

### 3.2 | Map of Misery

**FIGURE 4:**
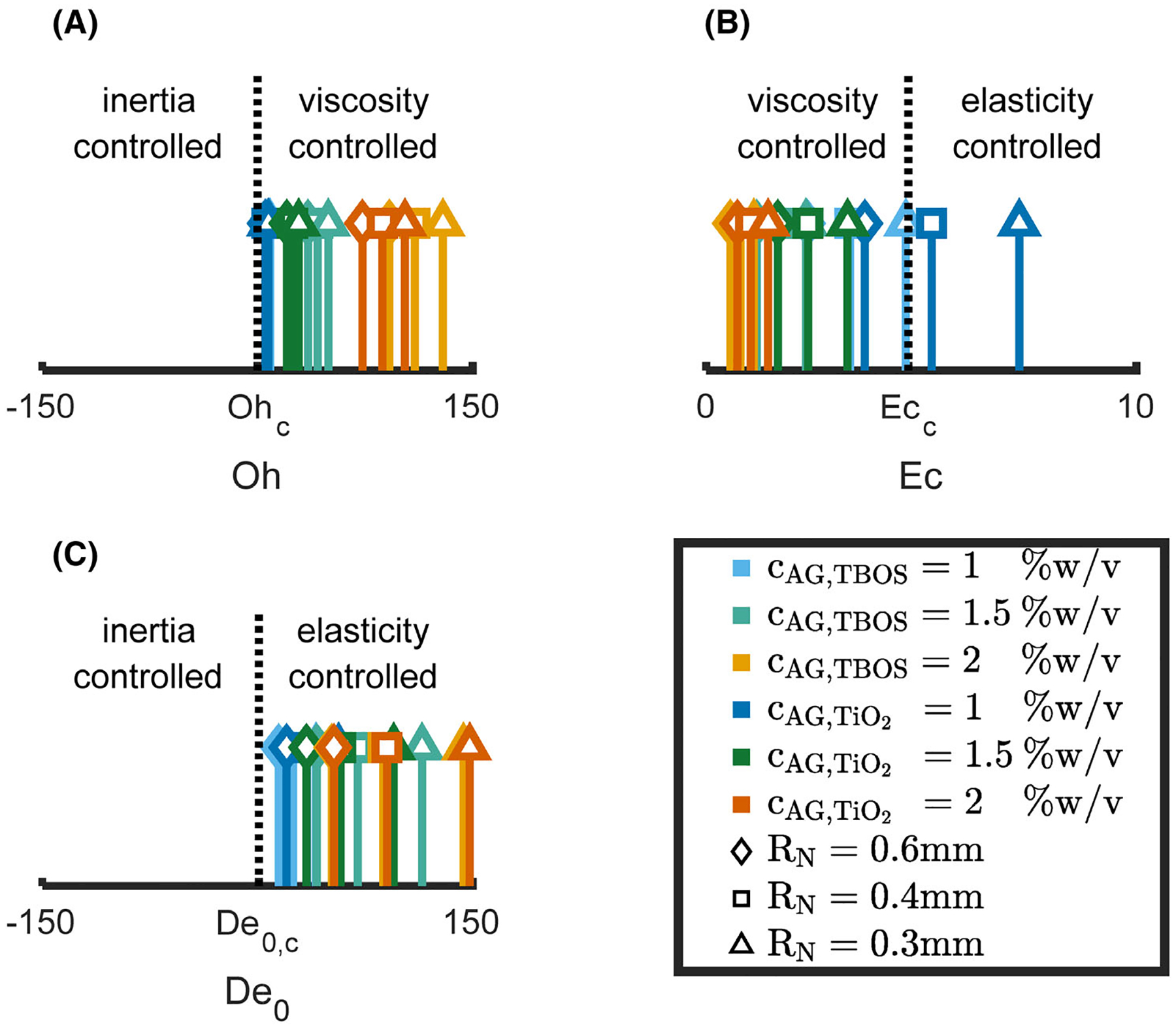
Material property dimensionless numbers Ohnesorge number (Oh), intrinsic Deborah number $\left(De_{0}\right)$, and elasto-capillary number (Ec), for the different fluids and nozzle diameters. (A) Oh values plotted in comparison to ${\text{Oh}}_{\text{c}}=0.2$. (B) Ec values plotted in comparison to ${\text{Ec}}_{\text{c}}=4.7$. (B) ${\text{De}}_{0}$ values plotted in comparison to ${\text{De}}_{0,\text{c}}=1$. AG, alginate; TBOS, tetrabutyl orthosilicate; ${\text{TiO}}_{2}$, titanium dioxide.

The Map of Misery^17^ global dimensionless groups, Oh, ${\text{De}}_{0}$, and Ec define the material properties of both fluid types at each concentration and nozzle size (Table 3). Comparisons of the material dimensionless number values to the critical value for each number, ${\text{Oh}}_{\text{c}},{\text{De}}_{0,\text{c}}$, and ${\text{Ec}}_{\text{c}}$ provide which material property (viscosity, inertia, or elasticity) dominates the thinning process (Figure 4). The properties dominating capillary thinning behavior were ranked, and it was determined that viscosity > elasticity > inertia. Thus, viscosity-controlled capillary thinning, and Ca was used as the dependent variable for the independent variables, perimeter and circularity of hydrogel beads.

We used the zero-shear viscosity, $\eta_{0}=\eta (\dot{\gamma }\to 0)$ as the viscosity parameter η in nondimensional groups Oh and Ec. Clasen et al. demonstrated with a polyisobutylene polymer in a pristane solvent, that weakly viscoelastic fluids with a rate dependent viscosity and $\eta_{0}$ behave similarly to Newtonian fluids with a viscosity equal to $\eta_{0}$.^17^ In addition, we assumed a constant density $\rho \sim 1000\;\text{kg}{\;\text{m}}^{-3}$ for all concentrations and both model fluids.

To compare elastic effects to inertial and viscous effects in Ec and ${\text{De}}_{0}$, we used the longest relaxation time obtained from shear $\tau_{\text{s}}$. With $\tau_{\text{s}}$ the majority of the fluids were found to be viscosity dominated with Ec < 4.7, with the exception of the model fluid $c_{\text{AG},{\text{TiO}}_{2}}=1\%\text{w}/\text{v}$ dispensed with radii $R_{\text{N}}=0.3$ and 0.4 mm which exceeded ${\text{Ec}}_{\text{c}}$. However, for semi-dilute and concentrated polymer solutions, timescales for the relaxation time correspond to a Rouse relaxation time based on molecular stretching, rather of the longest relaxation time of reptation of the polymer.^17,43,44^ We observed minimal effects of elasticity during capillary thinning in both DoS measurements. $\tau_{\text{E}}$ measured one to two orders of magnitude lower than $\tau_{\text{s}}$. Thus, $\tau_{\text{s}}$ overestimates elastic effects in capillary thinning, which can yield Ec > 4.7 and the values of Ec for $c_{\text{AG},{\text{TiO}}_{2}}=1\%\text{w}/\text{v}$ dispensed with radii $R_{\text{N}}=0.3$ and 0.4 mm are attributed to this overestimation. Therefore, we maintained that viscosity dominated capillary thinning and droplet formation. In conclusion, $\eta_{0}$ and $\tau_{\text{s}}$ provide the capability to rank the droplet formation dynamics of *concentrated* alginate solutions based on viscous, elastic, and inertial effects with shear rheology. However, further material characterization with extensional rheology may be necessary as $\tau_{\text{s}}$ can overestimate the elastic effects when evaluating polymer solutions in the semi-dilute and concentrated regimes.

### 3.3 | Bead perimeter and circularity as a function of capillary number

**FIGURE 5:**
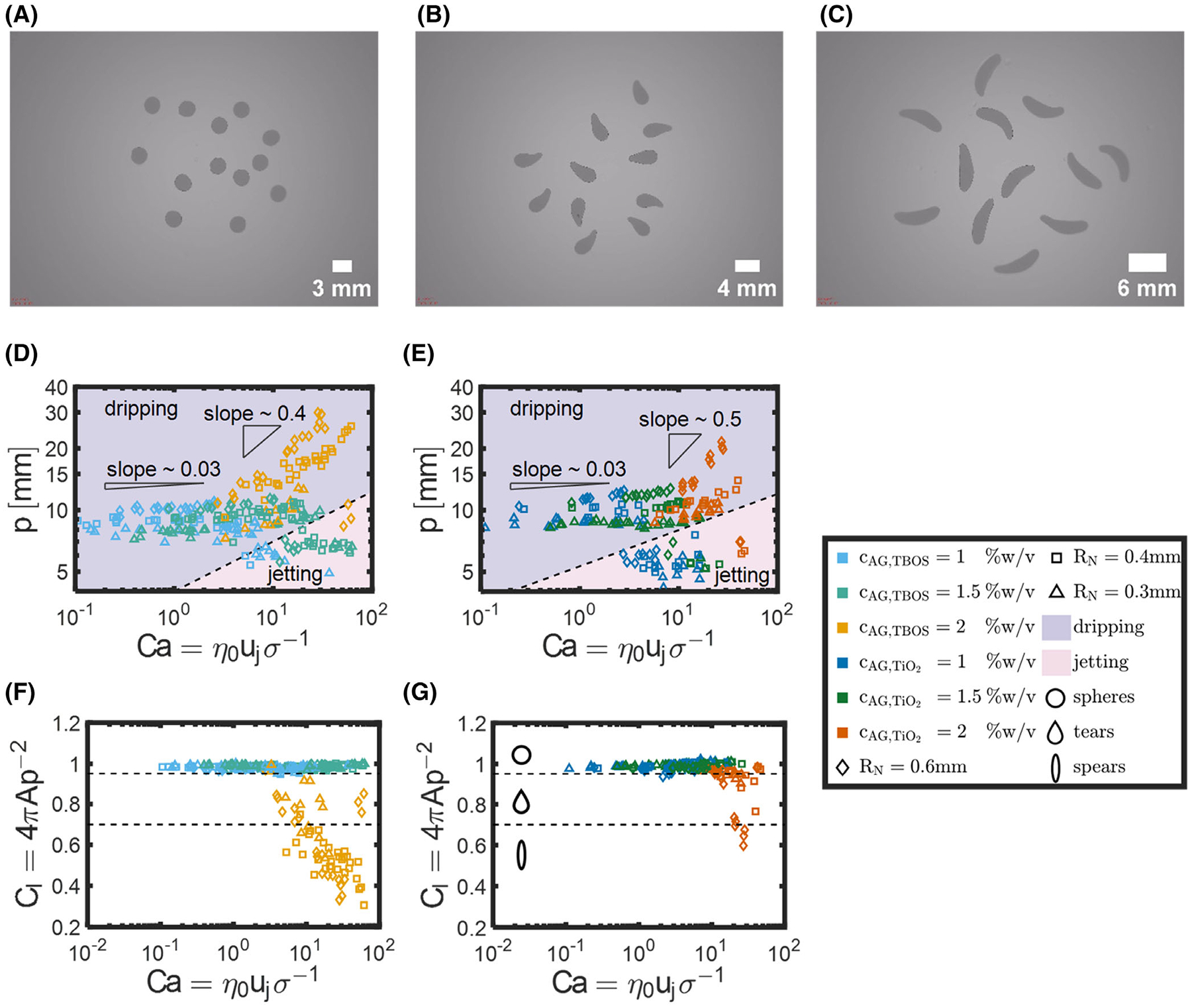
(A) Perimeter as a function of capillary number for $c_{\text{AG},\text{TBOS}}$. B Perimeter as a function of capillary number for $c_{{\text{AG},\text{TiO}}_{2}}$. (C) Circularity as a function of Capillary number for $c_{\text{AG},\text{TBOS}}$. (D) Circularity as a function of capillary number for $c_{{\text{AG},\text{TiO}}_{2}}$. For (A) and (B), right triangles represent the slope of perimeter with respect to Ca based on the concentrations. The slopes on the left-hand side represents $c_{\text{AG}}=\{1,1.5\}\%\text{w}/\text{v}$, whereas the slope on the right-hand side represents $c_{\text{AG}}=2\%\text{w}/\text{v}$. For (C) and (D), dotted lines represent the cutoffs for *spheres, tears, and spears*, represented by the circles, tears, and ellipse shapes on the left-hand side. For (D–G), points represent average perimeter or circularity for nine bead images $\left(n=9\right)$ for flow studies with an average Ca based on three measurements of volumetric flow rate $\left(n=3\right)$. Error bars were omitted for clarity as majority of the observed error lies within points. AG, alginate; TBOS, tetrabutyl orthosilicate; ${\text{TiO}}_{2}$, titanium dioxide.

Hydrogel beads of various sizes and shapes were generated based on the concentration of alginate in the model fluid and the flow parameters used in the bead generator. An example of hydrogel bead images demonstrates the *spheres, tears, and spears* obtained in these studies (Figure 5A–C). With image thresholding, we determined the perimeter p and area A of hydrogel beads using the Matlab Image Processing Toolbox *regionprops* function. Finally, the circularity $C_{\text{I}}$ was computed as:

(12) $$C_{\text{I}}=\frac{4\pi A}{p^{2}}$$

where A and p are the area and perimeter of the hydrogel bead, respectively. Note that based on this calculation, $C_{\text{I}}=1$ represents perfect circles and $C_{\text{I}}<1$ represents non-circular shapes, and shapes appear less like circles as $C_{\text{I}}$ decreases. *Spheres, tears, and spears* represent $C_{\text{I}}>0.95,0.7>C_{\text{I}}\geq 0.95$, and $C_{\text{I}}\leq 0.7$, respectively.

For both model fluids, the perimeter of hydrogel beads ranged between 4.2 and 30 mm (Figure 5D,E). In addition, the slope measured between the perimeter and Ca averaged 0.03 for $c_{\text{AG}}\leq 1.5\%\text{w}/\text{v}$ and all $R_{\text{N}}$. However, the average slope increased to 0.4 and 0.5 for $c_{\text{AG},\text{TBOS}}=2$ and $c_{\text{AG},{\text{TiO}}_{2}}=2\%\text{w}/\text{v}$, respectively, with $R_{\text{N}}=0.6\;\text{mm}$. The average slope for $c_{\text{AG},\text{TBOS}}=2$ and $c_{\text{AG},{\text{TiO}}_{2}}=2\%\text{w}/\text{v}$ was 0.4 ± 0.1 and 0.3 ± 0.2 for all $R_{\text{N}}$. For both model fluids, the circularity of hydrogels spanned 0.3 and 1.0 (Figure 5F,G). We observed spheres for all $c_{\text{AG}}$ and $R_{\text{N}}$. Tear and spear shaped hydrogel beads were only observed for ${\text{c}}_{\text{AG}}=2\%\text{w}/\text{v}$, with the exception of one case with $c_{\text{AG},{\text{TiO}}_{2}}=1\%\text{w}/\text{v}$ and $R_{\text{N}}=0.6\;\text{mm}$ with ${\text{C}}_{\text{I}}=0.94$.

Small increases in perimeter were observed for increasing Ca for both ${\text{c}}_{\text{AG}}=1\%\text{w}/\text{v}$ and 1.5% w/v, demonstrated by the triangle with a slope of 0.03. For $c_{\text{AG}}\leq 1.5\%\text{w}/\text{v},C_{I}\geq 0.95$, with only one exception. We observed a value of ${\text{C}}_{\text{I}}=0.94$ for ${\text{c}}_{\text{AG},\text{TiO}2}=1\%\text{w}/\text{v}$ and $R_{\text{N}}=0.6\;\text{mm}$, attributed to the impact of droplets on the surface of the crosslinking bath solution. In comparison, we observed the slope of perimeter as a function of Ca increase by an order of magnitude for model fluids with $c_{\text{AG}}=2\%\text{w}/\text{v}$. Specifically, we observed slopes of 0.4 and 0.5 for $c_{\text{AG},\text{TBOS}}=2\%\text{w}/\text{v}$ and $c_{\text{AG},{\text{TiO}}_{2}}=2\%\text{w}/\text{v}$ at $R_{\text{N}}=0.6\;\text{mm}$, respectively. In addition, $C_{\text{I}}$ for both model fluids with $c_{\text{AG}}=2\%\text{w}/\text{v}$ decreased rapidly with Ca, that led to tear and spear shapes. In general, increasing Ca increases p, due to the increase of the volume of droplets in as $u_{j}$ increases.^45^ Further, the increase of $\eta_{0}$ allows droplets to maintain non-spherical (tear and spear) shapes that occur with higher flow rates. Higher values of $\eta_{0}$ lead to greater resistance of the surface tension and drag forces from the air that shape the curvature of the droplet.^46^ Asadi et al. reported pear (tear) and prolate (spear) shapes occurring for alginate solutions with increasing $c_{\text{AG}}$, as well.^47^

The addition of a surfactant drives the differences observed in the values of p between the two model fluids. Higher values of p were observed on average for $c_{{\text{AG},\text{TiO}}_{2}}$ compared to $c_{\text{AG},\text{TBOS}}$ for Ca < 1. Zhang and Basaran showed that reducing the surface tension σ of water with a surfactant decreases the volume of droplets at low flow rates, but increases the volume of droplets at high flow rates, which could have a similar effect on our model fluids.^45^ However, the fluids studied here will behave differently than water, as inertia, rather than viscosity, controls the capillary thinning for water. Therefore, we observe p increase sharply for both fluids with respect to flow rate, with similar slopes.

Decreases in nozzle radius $R_{\text{N}}$ decreases p. Values of p measured at different $R_{\text{N}}$ followed similar trends but were shifted in the y-direction based on $R_{\text{N}}$ at the same capillary numbers. For example, p of $c_{\text{AG},\text{TBOS}}=1\%\text{w}/\text{v}$ at Ca = 1 were 2.6, 2.2, and 2.0 mm at nozzle sizes $R_{\text{N}}=0.6$, 0.4, and 0.3 mm, respectively. The reduction in p occurs as the volume of droplets depends proportionally on $R_{\text{N}}$, and further, reducing $R_{\text{N}}$ reduces the diameter of the filament thread that can break easier to perturbations.^45,48^ We observe similar trends between different values of $R_{\text{N}}$ because the characteristic velocity for viscosity-controlled thinning is constant and independent of $R_{\text{N}}$. In addition, the onset of jetting significantly reduced the perimeter of hydrogel beads in comparison to dripping regime. Dripping and jetting regimes occurred for all values of $c_{\text{AG}}$ and $R_{\text{N}}$, except for $c_{\text{AG}}=2\%\text{w}/\text{v}$ with nozzle radii $R_{\text{N}}=0.4$ and 0.3 mm, due to the maximum pressure available of the pressurized tank.

The onset of jetting appeared at different values of Ca based on $c_{\text{AG}}$ and $R_{\text{N}}$. Specifically, the value of Ca at the onset of jetting increased as $c_{\text{AG}}$ increased and $R_{\text{N}}$ decreased. Additionally, the onset of jetting increased the circularity of hydrogel beads with $c_{\text{AG}}=2\%\text{w}/\text{v}$; however, negligible differences were observed for lower concentrations. The interaction between Ca and $c_{\text{AG}}$ suggests that Ca depends on the shear-thinning index, n, as Clasen et al. demonstrated.^17^ We opted to maintain the zero-shear viscosity $\eta_{0}$ as we cannot determine the PL thinning velocity (obtained from the derivative of Equation 10) without capturing the filament thinning by high-speed imaging for every droplet experiment.

### 3.4 | Filament breakup and drop formation: high-speed imaging vs. simulation

**FIGURE 6:**
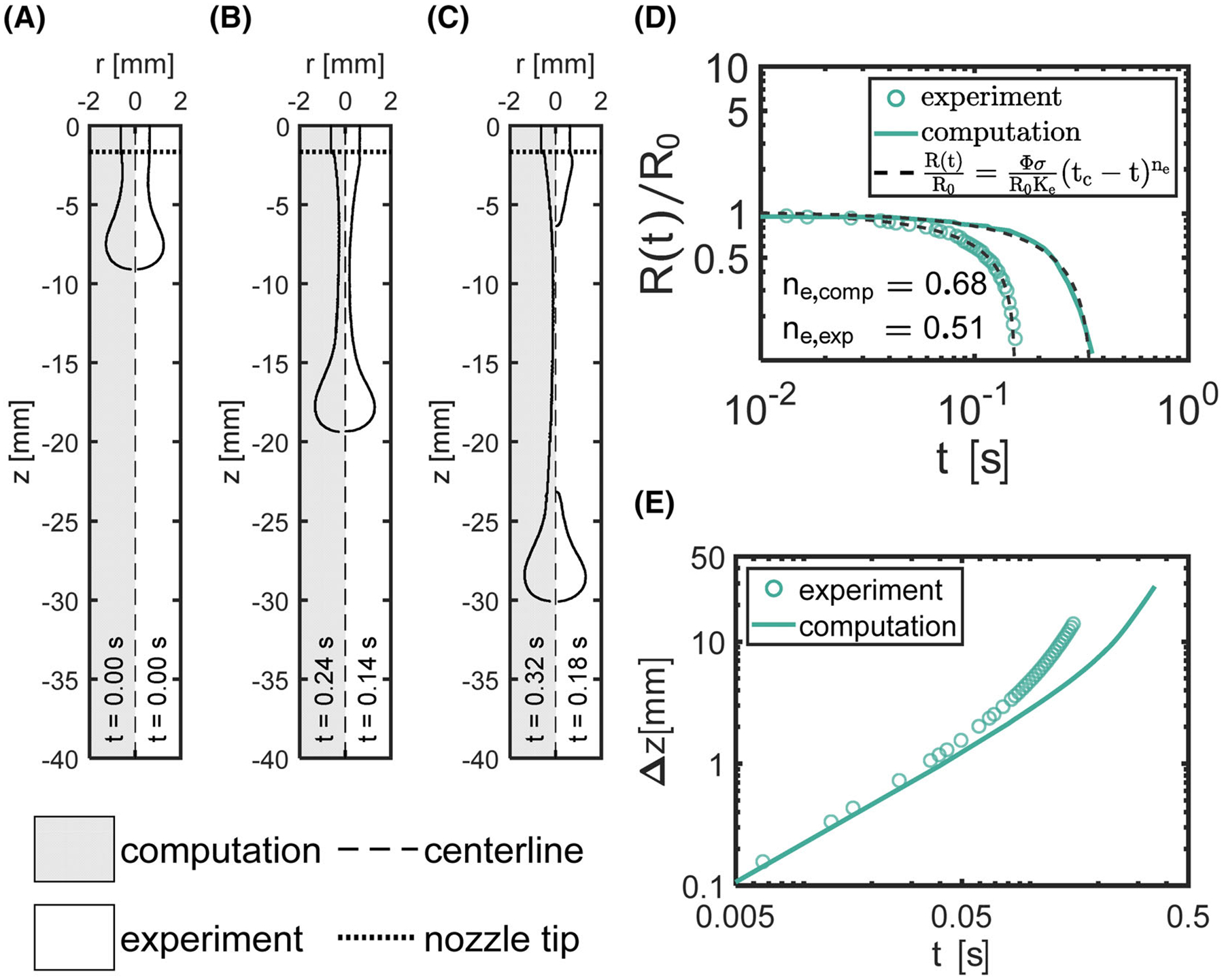
Comparison of numerical (gray background) against experimental (white background) results for the (A) initial, (B) intermediate, and (C) final stages of jet breakup and droplet formation. The dashed and dotted lines represent the centerline and nozzle tip, respectively. (D) Log–Log plot of $R(t)/R_{0}$ with respect to t. (E) Change in axial distance Δz with respect to t.

To corroborate values of $u_{j}$ determined from the pressured induced volumetric flow rate, we captured and analyzed high-speed footage, as well as simulated droplet formation via OpenFOAM for one model fluid, $c_{\text{AG},\text{TBOS}}=1.5\%\text{w}/\text{v}$ with one nozzle size $R_{\text{N}}=0.4\;\text{mm}$, within the dripping regime with $u_{j}=0.03{\;\text{ms}}^{-1}$. Computational and experimental results were compared based on qualitative and quantitative results. Three images that represent the initial, intermediate and final stages of jet breakup and droplet formation demonstrate the similarities and differences between computational and experimental results (Figure 6A–C). We compared PL models (Equation 10) fit to the normalized radius $R(t)/R_{0}$ results for experimental and computational data (Figure 6D). From these fits, we obtained exponential extension-thinning exponents, $n_{\text{e}}$, of 0.68 and 0.51 for computational and experimental results, respectively. In addition, we observed breakup at 0.16 and 0.36 s for experimental and computational results, respectively. Finally, we compared the change in axial distance $\Delta z=z-z_{0}$, where $z_{0}$ represents the initial axial distance, with respect to t for computational and experimental results (Figure 6E).

Qualitatively, we observed similarities between the high-speed imaging and computational results (Figure 6A–C). The droplet and filament shape appear to be similar between both computation and experimental results. However, we observed differences in the angle where the filament meets the droplet, with a sharper angle occurring for the experimental results in comparison to the computational results (Figure 6A). Others demonstrated slight differences in the droplet shapes observed between experimental and VOF model results.^29^ These differences likely occur as the interface depends on the computational mesh, with higher mesh sizes yielding greater inaccuracies in capturing sharp interfaces.^49^ In addition, we observed the fluid bulge at the nozzle tip for the experimental results after jet breakup. This occurs due to the recoil of the filament breaking and was observed for computational results as well after breakup occurred.

Quantitatively, we compared the filament thinning behavior between experimental and computational results (Figure 6D). First, the comparison of the normalized radius $R(t)/R_{0}$ as a function of time reveals the differences between the experimental and computational results. The extension-thinning indexes, $n_{e}$, obtained from PL models (Equation 10) fit to $R(t)/R_{0}$ indicates different extension-thinning behaviors between the results, as lower values of $n_{e}$ indicate greater thinning of viscosity in the presence of extension.^50^ Specifically, the values of $n_{e}$ were $n_{e}=0.68$ and 0.51 for computational and experimental results, respectively. Thus, $n_{\text{e},\text{comp}}=1.3n_{\text{e},\text{exp}}$. Further, the time for breakup $t_{\text{c}}$, for the computational result $\left(t_{\text{c},\text{comp}}=0.36\;\text{s}\right)$ is twice as long as the experimental results $\left(t_{\text{c},\text{comp}}=0.18\right)$. The differences between $n_{\text{e}}$ and $t_{\text{c}}$ presents two possibilities. (1) The alginate solutions are shear thinning, so the viscosity of the alginate solution is a function of the shear flow in the nozzle, resulting in both a reduced viscosity and entanglement density, as compared to its zero-shear viscosity, as it exits the nozzle. Therefore, the extensional flow of the filament depends on the shear imposed in the nozzle. Before the droplet volume accumulates to initiate extension, the viscosity will recover depending on the time for entanglements to return given by the maximum polymer relaxation time, a property of viscoelastic fluids. Because we excluded elasticity and only accounted for shear thinning in the computational fluid dynamics (CFD) model, the viscosity in the model can return instantaneously. Therefore, the model predicts a higher viscosity at the nozzle exit compared to the experiment, which results in nearly double the time for breakup. (2) The high viscosity ratio leads to inaccuracies predicting the correct amount of shear thinning. Hallmark et al. have reported low accuracy between experimental and computation models when capturing bubble behavior of an air bubble traveling through a viscous liquid at high viscosity ratios $\left(5.5\times 10^{5}<\lambda <3.9\times 10^{6}\right)$ while using a standard solver of OpenFOAM.^51^ Our computed viscosity ratio between the model fluid and air was 3.3 × 10^5^, which falls within the same order of magnitude of the viscosity ratios studied in Hallmark et al. Still, the filament thinning trends appear similarly, as the PL equation (Equation 10) fits both the experimental and computational results accurately. Therefore, an inclusion of a time-dependent term, or perhaps the use of a modified interface solver, could increase the accuracy of the simulations.

To corroborate the values of $u_{j}$ obtained from an average volumetric flow rate, we evaluated the change in the axial distance Δz with respect to time t between experimental high-speed imaging and simulation results (Figure 6E). As expected and similar to the results of $R(t)/R_{0},\;\Delta z$ deviates at later times, which can be explained by the values of $n_{e}$ for these results. For lower values of $n_{e}$, such as the experimental results compared to simulation results, the fluid resists extension less, and the filament would thin more rapidly. The differences likely occur due to the possibilities of either an effect of thixotropy or an inaccuracy from VOF methods for high viscosity ratios, as postulated above. However, again, the trends are similar between the computational and simulation results, overlapping at early times. Therefore, we found that the value of $u_{j}$ measured accurately captures the velocity of dispensed solutions and can be used to select bead perimeter and circularity for the ranges of material properties and operating conditions explored in this work.

## 4 | CONCLUSIONS

The operating conditions (nozzle radius $R_{\text{N}}$ and velocity $u_{j}$) based on the material properties of alginate solutions (viscosity $\eta_{0}$ and surface tension σ) that generate spherical, monodisperse millimeter-sized hydrogel beads were identified. Two model fluids composed of alginate were examined. One consisted of an emulsion of TBOS and alginate, with a reduced surface tension due to the addition of a surfactant and organic compound. The second fluid consisted of alginate and titanium dioxide used as an imaging agent to represent pure alginate solutions. From material property characterization and the use of the Map of Misery,^17^ we determined that, for both model fluids, $\eta_{0}$ dominates the resistance to the surface tension driven capillary thinning. We evaluated the size and shapes of beads, based on perimeter c and circularity $C_{\text{I}}$ captured from images of beads after crosslinking, with respect to $R_{N},u_{j},\eta_{0}$, and σ. Finally, it was corroborated that value of $u_{j}$ used for the dimensionless groups with high-speed imaging and computational data from an open-source CFD software, OpenFOAM, with the volume-of-method solver, *interFoam*.

While previous studies have evaluated the size and shape of hydrogel beads based on drop distance and concentration of alginate $c_{\text{AG}}$, this study provides the parameters necessary to generate desired hydrogel beads, within the ranges of the experiments presented, without calibration. In general, it was found that increases in Ca led to a gentle increase of p (slope ~O(-2)) with minimal changes to $C_{\text{I}}$ for $c_{\text{AG}}\leq 1.5\%\text{w}/\text{v}$. However, for $c_{\text{AG}}=2\%\text{w}/\text{v}$ this slope increased ~O(-1), demonstrating the interaction between $c_{\text{AG}}$ and Ca. Qualitative similarities between high-speed imaging and computational results were observed, such as the droplet shape and the trends in normalized radius and axial distance data with respect to time. Still, quantitative differences between these results were observed, indicating the need for a time-dependent term or more robust CFD models.

## Supplementary Material

Supplementary Material

Additional supporting information can be found online in the Supporting Information section at the end of this article.

## Figures and Tables

**TABLE 1:** Model fluids used to characterize the flow parameters of the bead generator.

Solution name	Alginate (AG) (% w/v)	Tetrabutyl orthosilicate (TBOS) (%w/v)	Span80 (% w/v)	Titanium dioxide (TiO_2_) (%w/v)
$c_{\text{AG},\text{TBOS}}=1\%\text{w}/\text{v}$	1	4.5	0.1	-
$c_{\text{AG},\text{TBOS}}=1.5\%\text{w}/\text{v}$	1.5	4.5	0.1	-
$c_{\text{AG},\text{TBOS}}=2\%\text{w}/\text{v}$	2	4.5	0.1	-
$c_{\text{AG},{\text{TiO}}_{2}}=1\%\text{w}/\text{v}$	1	-	-	0.1
$c_{\text{AG},{\text{TiO}}_{2}}=1.5\%\text{w}/\text{v}$	1.5	-	-	0.1
$c_{\text{AG},{\text{TiO}}_{2}}=2\%\text{w}/\text{v}$	2	-	-	0.1

**TABLE 2:** Shear and extensional rheology parameters. Parameters are displayed as the average and the standard deviation for three replicates $\left(n=3\right)$. A standard deviation with 0.00 indicates standard deviation <0.01.

	Shear rheology						
	Power law		Carreau-Yasuda				
Model fluid (% w/v)	$K_{\text{PL}}\left(\text{Pas}\right)$	$n_{\text{PL}}$	$\eta_{\infty }\left(\text{Pas}\right)$	$\eta_{0}\left(\text{Pas}\right)$	$K_{\text{CY,}\tau_{\text{s}}}\left(\text{ms}\right)$	$a_{\text{CY}}$	$n_{\text{CY}}$
$c_{\text{AG},\text{TBOS}}=1$	2.69 ± 0.30	0.58 ± 0.01	0.001	1.08 ± 0.13	30.0 ± 6.80	0.77	0.35 ± 0.03
$c_{\text{AG},\text{TBOS}}=1.5$	14.6 ± 0.86	0.44 ± 0.01	0.001	5.96 ± 0.92	82.9 ± 2.63	0.77	0.29 ± 0.20
$c_{\text{AG},\text{TBOS}}=2$	34.1 ± 0.32	0.38 ± 0.00	0.001	13.6 ± 0.11	99.2 ± 2.46	0.77	0.24 ± 0.00
$c_{\text{AG},{\text{TiO}}_{2}}=1$	2.85 ± 0.01	0.55 ± 0.00	0.001	1.12 ± 0.01	35.3 ± 1.86	0.77	0.34 ± 0.01
$c_{\text{AG},{\text{TiO}}_{2}}=1.5$	11.1 ± 0.99	0.44 ± 0.00	0.001	4.17 ± 0.29	59.9 ± 5.87	0.77	0.25 ± 0.02
$c_{\text{AG},{\text{TiO}}_{2}}=2$	37.7 ± 1.92	0.36 ± 0.00	0.001	14.3 ± 0.71	95.3 ± 3.81	0.77	0.21 ± 0.01
	Extensional rheology						
	PL		Shared parameters		EC		
Model fluid (% w/v)	$K_{\text{e}}\left(\text{Pas}\right)$	$n_{\text{e}}$	$\sigma \left({\text{mN}\;\text{m}}^{-1}\right)$	$t_{\text{c}}\left(\text{s}\right)$	$\tau_{\text{E}}\left(\text{ms}\right)$	$G_{\text{E}}\left(\text{Pa}\right)$	$\tau_{\text{E}}/\tau_{\text{S}}$
$c_{\text{AG},\text{TBOS}}=1$	6.61 ± 0.38	0.54 ± 0.01	50.6	0.10 ± 0.00	1.12 ± 0.22	0.70 ± 0.04	0.04
$c_{\text{AG},\text{TBOS}}=1.5$	11.8 ± 0.26	0.55 ± 0.01	49.0	0.22 ± 0.00	3.24 ± 0.50	0.84 ± 0.22	0.04
$c_{\text{AG},\text{TBOS}}=2$	23.1 ± 1.43	0.51 ± 0.01	48.1	1.39 ± 0.11	17.8 ± 2.50	0.62 ± 0.09	0.18
$c_{\text{AG},{\text{TiO}}_{2}}=1$	7.77 ± 0.39	0.51 ± 0.01	70.0	0.10 ± 0.01	1.05 ± 0.15	0.83 ± 0.05	0.03
$c_{\text{AG},{\text{TiO}}_{2}}=1.5$	16.1 ± 0.15	0.44 ± 0.01	69.6	0.44 ± 0.02	5.15 ± 0.89	1.03 ± 0.19	0.09
$c_{\text{AG},{\text{TiO}}_{2}}=2$	24.2 ± 1.19	0.46 ± 0.01	64.5	1.29 ± 0.21	19.6 ± 1.47	0.72 ± 0.17	0.21

Abbreviations: AG, alginate; EC, elasto-capillary; PL, power law; TBOS, tetrabutyl orthosilicate; ${\text{TiO}}_{2}$, titanium dioxide.

**TABLE 3:** Values of the material property dimensionless numbers Ohnesorge number (Oh), Intrinsic Deborah number $\left({\text{De}}_{0}\right)$, and elasto-capillary number (Ec), for the different fluids and nozzle diameters.

	$\text{Oh}\left({\text{Oh}}_{\text{c}}=0.2\right)$			${\text{De}}_{0}\left({\text{De}}_{0,\text{c}}=1\right)$			$\text{Ec}\left({\text{Ec}}_{\text{c}}=4.7\right)$		
	$R_{\text{N}}\left(\text{mm}\right)$			$R_{\text{N}}\left(\text{mm}\right)$			$R_{\text{N}}\left(\text{mm}\right)$		
Model fluid (% w/v)	0.6	0.4	0.3	0.6	0.4	0.3	0.6	0.4	0.3
$c_{\text{AG},\text{TBOS}}=1$	6.3	7.5	8.8	2.4	3.3	4.6	14.7	24.9	40.9
$c_{\text{AG},\text{TBOS}}=1.5$	35.2	42.0	49.5	1.2	1.7	2.3	41.4	70.1	115
$c_{\text{AG},\text{TBOS}}=2$	92.1	110.	129.	0.6	0.8	1.1	51.8	87.7	144
$c_{\text{AG},{\text{TiO}}_{2}}=1$	5.5	6.6	7.8	3.7	5.2	7.3	20.4	34.6	56.7
$c_{\text{AG},{\text{TiO}}_{2}}=1.5$	20.6	24.5	28.9	1.7	2.4	3.3	34.3	58.0	95.0
$c_{\text{AG},{\text{TiO}}_{2}}=2$	73.2	87.2	103	0.7	1.0	1.4	53.4	90.4	148

Abbreviations: AG, alginate; TBOS, tetrabutyl orthosilicate; ${\text{TiO}}_{2}$, titanium dioxide.

## Data Availability

All data are available on the Fogg Lab github website: https://github.com/fogg-lab/Spheres-Tears-Spears. The rawdata_flowsweeps and rawdata_DoS folders contain all shear and extensional rheology data, respectively, plotted in Figure 3 and reported in Table 2. The rawdata_bdgen_mm/rawdata_PT_dotV.xlsx file contains all pressure and flow rate data for all trials plotted in Figure 5. The rawdata_bdgen_mm/rawdata_mm_im.xlsx file contains all nondimensional numbers, perimeter, and circularity data for Figures 4 and 5 and Table 3. The rawdata_bdgen_mm/rawdata_sften.xlsx file contains all surface tension data reported in Table 2. The rawdata_hsimg_comp folder contains the high-speed imaging and simulation videos, as well as the radius and time data extracted from the videos, used to generate Figure 6. The rawdata_DOE folder contains the data for Figures S1 and S2. Lastly, the rawdata_freqsweeps folder contains the data used in Figure S4. Rheological measurements (shear and extensional) were performed in triplicates $\left(n=3\right)$, where independent samples were tested for each measurement. The average ± standard deviation is documented in Table 2. Points in Figure 3A represent the average between triplicates, and points in Figure 3B are shown as an example of a single experiment. Error bars in Figure 3 are not shown as the error bars fall within the boundaries of the points shown. The values of dimensionless groups (Table 3 and Figure 4) were calculated from the averaged viscosity and relaxation time values in Table 2. For Figure 5D–G, points represent the average of perimeter or circularity for nine bead images (n=9) from independent samples with three measurements of volumetric flow rate $\left(n=3\right)$. Error is not shown as the error bars fall with the points on the plot. Figure 6 represents data from a single high-speed video and a single simulation. Data in Figure S1 represents Reynolds numbers calculated from three measurements of volumetric flow rate $\left(n=3\right)$. Figure S2 represents models obtained from design of experiments empirical models. Figure S3 demonstrates selection of nine replicates used for bead images to generate the operating space in Figure 5D–G, plotted as the ratio of the mean perimeter with variable replicates to the mean perimeter with 15 replicates. Note that nine replicates were used as all bead images contained at least nine hydrogel beads, and not all images included up to 15 hydrogel beads. Figure S4A points represent values of elastic and viscous modulus for a single independent sample at variable concentrations. Figure S4B compares the values of the relaxation time measured as the crossover point between the elastic and viscous from the oscillatory tests in Figure S4A to relaxation times calculated from the shear rheological tests in Figure 3A.

## NOMENCLATURE

A: area of bead
$a_{\text{C}\text{Y}},K_{CY}$, and $n_{\text{C}\text{Y}}$: Carreau–Yasuda model parameters
AG: alginate
$A_{\text{N}}$: cross-sectional area of nozzle
C: courant number
Ca: capillary number
$c_{\text{AG}}$: concentration of alginate
$c_{\text{AG,TBOS}}$: model fluid (TBOS)
$c_{\text{A}\text{G},{\text{T}\text{i}\text{O}}_{2}}$: model fluid (TiO_2_)
$C_{\text{I}}$: circularity of bead
D: rate-of-deformation tensor
${\text{D}\text{e}}_{0}$: intrinsic Deborah number
Ec: elasto-capillary number
FPS: frames per second
$G_{\text{E}}$: elastic modulus
$K_{\text{e}}$: power law prefactor coefficient in extension
$K_{\text{P}\text{L}}$ and $n_{\text{P}\text{L}}$: power law model parameters
$n_{\text{e}}$: power law exponent in extension
Oh: Ohnesorge number
p: perimeter of bead
$R_{O}$: outer diameter
$R_{0}$: initial radius of the filament
Re: Reynolds number
$R_{\text{N}}$: inner nozzle radius
R(t): time-dependent radius of the filament
$R(t)/R_{0}$: normalized radius
t: time
TBOS: tetrabutyl orthosilicate
$t_{\text{c}}$: time of onset of the elasto-capillary regime
$t_{f}$: pinch-off time
${\text{T}\text{i}\text{O}}_{2}$: titanium dioxide
u: velocity vector
$u_{j}$: jet velocity
$u_{r}$: compressive velocity in the interface region
$\dot{V}$: volumetric flow rate
We: Weber number
Wi: Weissenberg number
z: axial distance
$z_{0}$: initial axial distance
α: volume fraction of liquid
$\dot{\gamma }$: shear rate
Δt: time step
Δx: side length of quadrilateral cells
Δz: change in axial distance
η: viscosity
$\eta_{\infty }$: infinite shear viscosity
$\eta_{0}$: zero-shear viscosity
λ: viscosity ratio
$\mu_{\text{air}}$: viscosity of air
ρ: density
σ: surface tension
τ: relaxation time (general)
$\tau_{\text{E}}$: relaxation time (extensional)
$\tau_{\text{liquid}}$: stress tensor associated with the liquid
$\tau_{\text{S}}$: relaxation time (shear)
Φ: prefactor constant dependent on the power law exponent in extension
