# Supplementary material for: Spheres, tears, and spears: Regulating the perimeter and circularity of millimeter-sized alginate hydrogel beads
Source: AIChE J. Author manuscript; Available in PMC 2026 Apr 9. (PMC13061411; doi:10.1002/aic.70279)
Supplement: Supplementary Material [file NIHMS2149140-supplement-Supplementary_Material.docx]

Supplementary Information

1. Evaluation of a coaxial air device, piezoelectric ring, and Reynolds number using statistical optimization via Design of Experiments

We used model fluids comprised of sodium alginate at different concentrations ($c_{AG}\in\left\{ 1,1.5,2 \right\}$\ (%w/v)), tetrabutoxysilane (TBOS) at 5% v/v, and Span80 at 0.1% v/v, in water to characterize the flow parameters of the bead generator with a coaxial air device and piezoelectric ring oscillator for one nozzle size $R_{N}=0.4$ mm. With the density of the model fluid, $\rho$, the velocity of the jet, $u$, the radius of the nozzle, $R_{N}$, and the zero shear viscosity of the model fluid, $\eta_{0}$, we identified flow ranges in terms of the Reynolds numbers ($Re= \left( \frac{inertial forces}{viscous forces} \right)=\frac{\rho uR_{N}}{\eta0})$ based on the inlet pressure for flow, $P$ (**Fig. SI1**) .

**
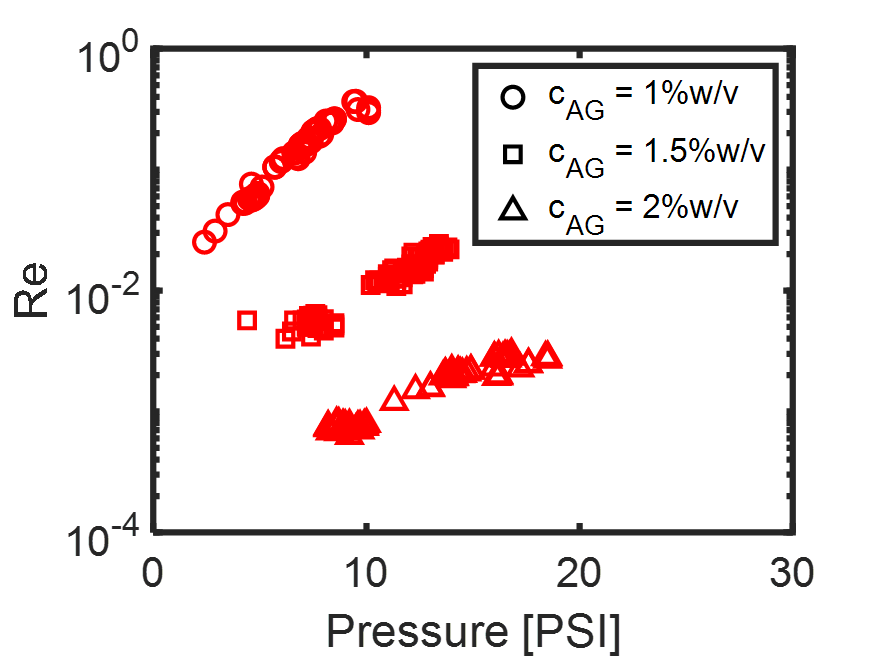
**

**Fig. SI1** Reynolds number as a function of alginate concentration and tank pressure used in DOE.

From this data, we used the range of Reynolds number specific to $c_{AG}$ to identify the effects of the independent variables ($Re$, vibrational frequency $f$, and air flow $Q_{a}$), on the dependent variables (bead perimeter $p$ and circularity $C_{I}$) with a Design of Experiments (DOE) approach. The ranges for $Re$ depend on $c_{AG}$ (**Fig. SI1**). Otherwise, $f$ ranged between 0.2 and 2 kHz, and $Q_{a}$ ranged between 0 and 5 L/min. We used a digital microscope to capture images of beads. Image analysis was used to determine $p$ and $C_{I}$. The perimeter was measured as a continuous boundary around each bead. $C_{I}$ was determined based on the equation:

$C_{I}=\frac{4\pi A}{p^{2}}$ (**Eqn. SI1**)

Where $A$ is the area determined from image analysis. As such, $C_{I}$ ranges from 0 to 1, where a value of 1 represents a perfect circle.

From the results we generated second-order polynomial response surface equations to analyze the effects of the input factors ($Re$, vibrational frequency $f$, and air flow $Q_{a}$) to their responses (bead perimeter $p$ and circularity $C_{I}$). Multiple linear regression was used to obtain predictive models for each response variable. Note that experimental data was obtained first and used to fit models. Singular value decomposition was used to obtain regression coefficients. Analysis of variance (ANOVA) tests were used to determine the statistical analysis of the experimental data and regression coefficients, and to obtain interactions between the variables and the responses. Finally, factor effect plots were used to compare the individual contributions of each factor on each of the responses measured. Each input factor was varied over its specific range while all the other input factors were held constant at their averages. Note that coded units were used to evaluate the factor effects and were built using the following example equation:

$x=\frac{(x-x_{span} )}{x_{mid}}$ (**Eqn. SI2**)

Where $x$, $x_{mid}$, and $x_{span}$ represent the independent variable, the midpoint of the range selected for the independent variable, and the span of the independent variable which is determined by subtracting the midpoint of the range by the maximum of the range. Therefore, the minimum, midpoint, and maximum of the variables in coded units are -1, 0, and +1.

From the factor effects, we observe that $p$ and $C_{I}$ depend on all three factors, however, the $Re$ dominates the behavior of $p$ and $C_{I}$ (**Fig. SI2**). This is shown by the range of predicted values for $p$ and $C_{I}$ as a function of $Re$ in comparison to $f$ and $Q_{a}$. One exception occurs for $C_{I}$ with $c_{AG}=$ 1 %w/v, that is most effect by $Q_{a}$, however, note that the range of plots are exceedingly small (0.980 – 0.986), and therefore, $C_{I}$ remains relatively constant for $c_{AG}=$ 1 %w/v.

**Fig. SI2** Factor effect plots of **A** circularity and **B** perimeter with respect to Reynolds number $Re$, piezoelectric ring frequency $f$, and air flow rate $Q_{a}$ for concentration of alginate $c_{AG}=1$ %w/v. Factor effect plots of **C** circularity and **D** perimeter with respect to $Re$, $f$, and $Q_{a}$ for concentration of alginate $c_{AG}=1.5$ %w/v. Factor effect plots of **E** circularity and **F** perimeter with respect to $Re$, $f$, and $Q_{a}$ for concentration of alginate $c_{AG}=2$ %w/v.

1. Selection of the number of replicates used for perimeter and circularity measurements

**Fig. SI3** **A** Box and whisker plot of the ratio of the mean perimeter with variable replicates n = x to mean perimeter with 15 replicates n= 15. **B** Box and whisker plot of the ratio of the standard deviation of perimeter with variable replicates n = x to the standard deviation of perimeter with 15 replicates n= 15.

The mean and standard deviation of the perimeter measured for hydrogels was collected for all images that contained at least 15 hydrogel beads (127 images). Comparisons were made of the mean and standard deviation at 5 different counts of replicates ($n=3, 6, 9, 12, 15$) against the mean and standard deviation at 15 replicates (**Fig. SI3**). 9 replicates provided upper and lower quartiles within 2.7% and 13% of the mean and standard deviation, respectively, observed at 15 replicates. In contrast, 3 replicates provided upper and lower quartiles within 8.3% and 58% of the mean and standard deviation, respectively, observed at 15 replicates. Therefore, we selected 9 replicates, as the values approach those of higher replicates, and all images contained at least 9 hydrogel beads.

1. Relaxation time of solutions obtained from dynamic oscillatory shear

Dynamic oscillatory shear measurements were performed on an ARG2 rheometer (TA instruments, New Castle, Delaware, USA) with a 60mm 1° cone top geometry and a Peltier plate bottom geometry. Oscillatory experiments were conducted on model fluids with tetrabutyl orthosilicate, TBOS, at $c_{TBOS}=$ 4.5 %w/v and $c_{AG}\in$ {1, 2, 3} %w/v to validate the characteristic relaxation time, $\lambda$, obtained from Carreau-Yasuda equations. The samples were subjected to oscillatory frequencies ramped logarithmically from $\omega=$ [1,500] rad s^-1^ with a strain amplitude $\gamma=$ 5% with the resultant elastic ($G^{'}$) and viscous ($G^{''}$) modulus measured. The Deborah number ($De$) can be used to determine $\lambda$ at the crossover frequency, $\omega_{c}$, where the moduli become equal in value, $G^{'}=G^{''}$ and $De \sim O(1)$ :

$De=\frac{\lambda}{\omega_{c}}\sim O\left( 1 \right)\Rightarrow\lambda=\omega_{c}$ (**Eqn. SI3**)

**Fig. SI4** **A** Elastic ($G^{'}$) and viscous ($G^{''}$) modulus as a function of oscillatory frequency $\omega$. Circles, triangles, and dotted lines represent $G^{'}$, $G^{''}$, and extrapolated $G^{'}$. Blue, magenta, and yellow represent $c_{AG}$ of 1, 2, and 3 %w/v, respectively. **B** Relaxation time $\lambda$ as a function of $c_{AG}$ based on the determination from oscillatory shear or slow sweeps, represented by circles and squares, respectively.

Oscillatory frequency sweeps were conducted to measure and compare the relaxation time determined from flow sweeps for the model fluid at three different sodium alginate concentrations $C_{AG}\in\left\{ 1, 2, 3 \right\} \backslash[g/dL]$ (**Fig. SI4A**). The relaxation time was taken where the elastic modulus crosses the viscous modulus, i.e., $G^{'}=G^{''}$. The relaxation time increased with an increase in sodium alginate concentration. The relaxation time was nearly equal between the different measurements (**Fig. SI4B**). Thus, we used relaxation times determined from steady shear flow sweep experiments throughout the manuscript.

1. Pendant drop measurements to determine interfacial tension of solutions

Pendant drop experiments were conducted on an FTA 135 Contact Angle Goniometer (First Ten Angstroms, Inc., Newark, CA, USA). Pendant drop experiments were performed on model fluid solutions to determine the interfacial tension $\sigma$ at the air-liquid interface. Liquid was gently dispensed from a blunt edge nozzle to form a droplet and the end of the nozzle (**Fig. SI5**). The droplet was imaged, and the interfacial tension was calculated based on the geometry of the particle described in Berry et al.^1^ The Worthington number ($Wo=\frac{\Delta\rho gV_{d}}{\pi\sigma D_{n}}$) was calculated for each experiment to characterize the measurement precision (accurate $\gamma$ when $Wo\sim1$); where $\Delta\rho$ is the difference in density between the liquid and air, $g$ is the acceleration due to gravity, $V_{d}$ is the volume of the droplet suspended, $\sigma$ is the interfacial tension measured, and $D_{n}$ is the diameter of the nozzle.


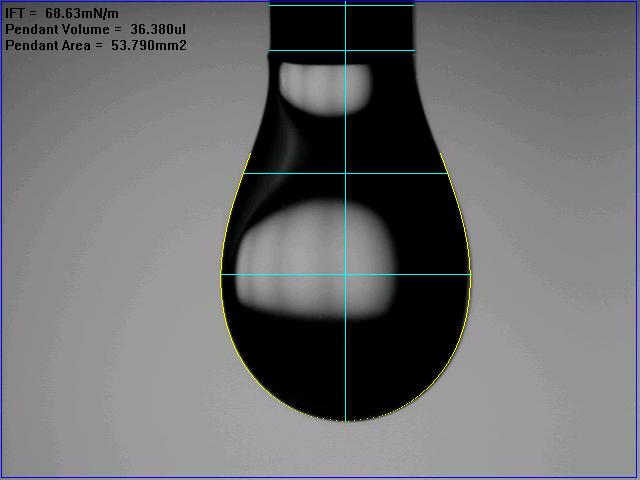


**Fig. SI5** Example of pendant drop with calculated interfacial tension, pendant volume, and pendant area.

1. Utilizing the operating space to generate hydrogel beads

From **Figure 5** in the main text, users can produce hydrogel beads with a specific bead perimeter and circularity, given that they lie within the operating space in **Figure 5**, based on the material properties, viscosity and surface tension, and operating parameters, nozzle radius and jet velocity. For example, let’s generate an alginate hydrogel bead with TBOS with concentrations of 1.5, 4.5, and 0.1 %w/v for alginate, TBOS, and Span80, respectively. Viscosity and surface tension should be measured using shear rheology (**Section 2.2.1** in the main text) and pendant drop (**SI4**) methods, respectively. This results in a zero-shear viscosity $\eta_{0}=6.0$ Pa s and surface tension $\sigma=49.0$ mN m^-1^.

Now that material properties have been determined; we can move onto operating conditions. Within the operating space, a bead size (perimeter $p$) and shape (circularity $C_{I}$) can be selected based on the Capillary number $Ca=\eta_{0}u_{j}\sigma^{-1}$. Note that for circular beads ($C_{I}>0.95$), $p$ can be translated to a diameter $D=p/\pi$. We find beads that exist in the operating space that suit our criteria. For this example, we will select circular beads $C_{I}>0.95$ with $p=10$ mm ($D=3.2$ mm) from our operating space (**Fig. SI6A,B**). The nozzle radius $R_{N}$ controls $p$, such that $p\propto R_{N}$, but has little to no effect on $C_{I}$ for $\eta_{0}\leq6.0$. For this case, to achieve $p=10$ mm, we select $R_{N}=0.6$ mm, or a 16-gauge nozzle, and $Ca=0.9$. The last remaining variable to control is $u_{j}$. With $Ca=0.9$, we can calculate the required value of $u_{j}$.

$u_{j}=\frac{Ca}{\eta_{0}}\sigma=\frac{0.9}{6.0 Pa s}0.049 Nm^{-1}=0.0074 m s^{-1}=7.4 mm s^{-1}.$ (**Eqn. SI4**)

The value of $u_{j}$ represents the average velocity within the nozzle before exiting

$u_{j}=\dot{V}/A_{N}$ (**Eqn. SI5**)

where $\dot{V}$ and $A_{N}=\pi R_{N}^{2}$ represent the volumetric flow rate and area of the nozzle respectively. Therefore, one can set a value of $V$ by measuring volume over time at specific pressures in our bead generator apparatus, or by setting a value on a syringe pump. For our case:

$\dot{V}=u_{j}A_{N}=u_{j}\pi R_{N}^{2}=7.4 mm s^{-1}\times\pi\times\left( 0.6 mm \right)^{2}=8.4 mm^{3} s^{-1}=30 mL hr^{-1}.$ (**Eqn. SI6**)

Pumping an alginate-TBOS solution with $c_{AG,TBOS}=1.5$ %w/v at $\dot{V}=30$ mL hr^-1^ through a nozzle with $R_{N}=0.6$ mm will result in beads shown in **Fig. SI6C**.

Steps to generate bead:

1. Select solution
   1. Measure zero-shear viscosity $\eta_{0}$ (**Section** **2.2.1** in the main text)
      1. For spherical beads, we recommend $\eta_{0}\leq6$ Pa s
   2. Measure surface tension $\sigma$ (**SI4**)
2. Select bead perimeter and circularity from operating space for similar material properties at a given Capillary number $Ca$ and nozzle radius $R_{N}$
   1. Determine velocity $u_{j}$ from $Ca$, $\eta_{0}$, and $\sigma$ (**Eqn. SI4**)
   2. Determine volumetric flow rate $\dot{V}$ from $u_{j}$ and $R_{N}$ (**Eqn. SI6**)
3. Generate beads using $\dot{V}$ and $R_{N}$ with solution.

**Fig. SI6** Selection of **A** bead perimeter $p$ and **B** circularity $C_{I}$ used for sample calculation. Here we select $p=10$ mm and $C_{I}=0.97$ with $c_{AG,TBOS}=1.5$ %w/v and $R_{N}=0.6$mm. To achieve the selected values of $p$ and $C_{I}$, $Ca=0.9$. **C** The resultant beads with the selected material properties and operating parameters.

**References**

(1) Berry, J. D.; Neeson, M. J.; Dagastine, R. R.; Chan, D. Y. C.; Tabor, R. F. Measurement of Surface and Interfacial Tension Using Pendant Drop Tensiometry. *J. Colloid Interface Sci.* **2015**, *454*, 226–237. https://doi.org/10.1016/j.jcis.2015.05.012.
